# Whither the etiopathogenesis (and scoliogeny) of adolescent idiopathic scoliosis? Incorporating presentations on scoliogeny at the 2012 IRSSD and SRS meetings

**DOI:** 10.1186/1748-7161-8-4

**Published:** 2013-02-28

**Authors:** R Geoffrey Burwell, Peter H Dangerfield, Alan Moulton, Theodoros B Grivas, Jack CY Cheng

**Affiliations:** 1 Centre for Spinal Studies and Surgery, Nottingham University Hospitals Trust, Queen’s Medical Centre Campus, Derby Road, Nottingham, NG7 2UH, UK; 2 University of Liverpool, Ashton Street, Liverpool, L69 3GE, UK; 3 Staffordshire University, Leek Road, Stoke-on-Trent, ST4 2DF, UK; 4 Royal Liverpool Children’s Hospital, Eaton Road, Liverpool, L12 2AP, UK; 5 Department of Orthopaedic Surgery, King’s Mill Hospital, Sutton Road, Mansfield, NG17 4JL, UK; 6 Department of Trauma and Orthopaedics, “Tzanio” General Hospital, Tzani and Afendouli 1 st, Piraeus, 18536, Greece; 7 Department of Orthopaedics & Traumatology, The Joint Scoliosis Research Centre of the Chinese University and Nanjing University, Prince of Wales Hospital, The Chinese University of Hong Kong, Hong Kong, SAR, China

**Keywords:** Scoliosis, Etiology, Pathogenesis, Scoliogeny, Epigenetics

## Abstract

This paper aims to integrate into current understanding of AIS causation, etiopathogenetic information presented at two Meetings during 2012 namely, the International Research Society of Spinal Deformities (IRSSD) and the Scoliosis Research Society (SRS). The ultimate hope is to prevent the occurrence or progression of the spinal deformity of AIS with non-invasive treatment, possibly medical. This might be attained by personalised polymechanistic preventive therapy targeting the appropriate etiology and/or etiopathogenetic pathways, to avoid fusion and maintain spinal mobility. Although considerable progress had been made in the past two decades in understanding the etiopathogenesis of adolescent idiopathic scoliosis (AIS), it still lacks an agreed theory of etiopathogenesis. One problem may be that AIS results not from one cause, but several that interact with various genetic predisposing factors. There is a view there are two other pathogenic processes for idiopathic scoliosis namely, initiating (or inducing), and those that cause curve progression. Twin studies and observations of family aggregation have revealed significant genetic contributions to idiopathic scoliosis, that place AIS among other common disease or complex traits with a high heritability interpreted by the genetic variant hypothesis of disease. We summarize etiopathogenetic knowledge of AIS as theories of pathogenesis including recent multiple concepts, and blood tests for AIS based on predictive biomarkers and genetic variants that signify disease risk. There is increasing evidence for the possibility of an underlying neurological disorder for AIS, research which holds promise. Like brain research, most AIS workers focus on their own corner and there is a need for greater integration of research effort. Epigenetics, a relatively recent field, evaluates factors concerned with gene expression in relation to environment, disease, normal development and aging, with a complex regulation across the genome during the first decade of life. Research on the role of environmental factors, epigenetics and chronic non-communicable diseases (NCDs) including adiposity, after a slow start, has exploded in the last decade. Not so for AIS research and the environment where, except for monozygotic twin studies, there are only sporadic reports to suggest that environmental factors are at work in etiology. Here, we examine epigenetic concepts as they may relate to human development, normal life history phases and AIS pathogenesis. Although AIS is not regarded as an NCD, like them, it is associated with whole organism metabolic phenomena, including lower body mass index, lower circulating leptin levels and other systemic disorders. Some epigenetic research applied to Silver-Russell syndrome and adiposity is examined, from which suggestions are made for consideration of AIS epigenetic research, cross-sectional and longitudinal. The word *scoliogeny* is suggested to include etiology, pathogenesis and pathomechanism.

## Review

This paper aims to integrate into current thinking about AIS causation, etiopathogenetic information presented at two Meetings during 2012 namely, the International Research Society of Spinal Deformities (IRSSD) and the Scoliosis Research Society (SRS). This is done by arranging extracts of selected presentations as paragraphs (with bold italicised headings) into the structure of an invited review article published at that IRSSD Meeting beginning with the Abstract [1]. These placements reveal where progress is being made and suggest fields where the focus is becoming clearer, needs enlarging, or is neglected. There is a lack of such collated updated preliminary etiopathogenetic researches in the literature. No attempt is made here to provide level of evidence for the selected presentations - most of which are not full papers. Nor are the findings interpreted with respect to theories of curve initiation, progression, or the consequence of having AIS, other than that provided by their authors. Readers are invited to apportion significance to those researches that interest them.

## Terminology

The word *etiology* strictly means the factor(s) causing the AIS, *pathogenesis* mode of origin of the morbid process, and *pathomechanism* sequence of events in the evolution of its structural and functional changes that result from the pathological process [2]. The word *etiopathogenesis* is used to embrace etiology and pathogenesis. We suggest the word *scoliogeny* as the collective noun to include etiology, pathogenesis and pathomechanism.

## Introduction

Research into the causation of adolescent idiopathic scoliosis (AIS) draws heavily from mechanical and biological disciples, but still lacks an agreed theory of etiopathogenesis [3-10]. Genetic factors are believed to play an important role in the etiology of AIS with considerable heterogeneity [5,6]. The research problem is complicated by the suspicion that AIS may result not from one cause, but several that interact [3,9]. Genetic, and now genomic, research on AIS have not yet provided the therapeutically-required etiologic understanding. In other diseases and particularly diseases of developmental origin [11] and late-onset chronic non-communicable diseases (NCDs) [12], research on the role of environmental factors and *epigenetics* after a slow start has exploded in the last decade [12-17]. Not so for AIS research and the environment where, except for monozygotic twin studies, there are only sporadic reports suggesting that environmental factors are at work in etiology [18]. Epigenetics, a relatively recent field, evaluates factors concerned with gene expression in relation to environment, disease, normal development and aging, with a complex regulation across the genome during the first decade of life. Elsewhere we commented on etiopathogenetic concepts as they may relate to normal spine development and AIS pathogenesis [18]. Here we consider:

(1) some theories of pathogenesis including recent multiple concepts;

(2) blood tests for AIS based on predictive biomarkers and genetic variants that signify disease risk;

(3) epigenetic concepts as they may relate to human development and life history phases;

(4) AIS linked to the aging process as a non-communicable disease (NCD);

(5) findings from epigenetic methods applied to the Silver-Russell syndrome and adiposity; and

(6) suggestions for applying epigenetic methods to AIS etiopathogenesis in cross-sectional and longitudinal studies.

## Etiopathogenesis 2011

In a review of Top Theories of AIS Wang *et al.*[5] concluded:

• considerable progress had been made in the past two decades in understanding the etiopathogenesis of AIS;

• current knowledge is still fragmented;

• we are still far from understanding fully the different etiopathogenetic pathways and mechanisms for example –

• the general skeletal and relative anterior spinal overgrowth (RASO) of AIS girls have not been related securely to endocrinology;

• the abnormal extra-spinal skeletal length asymmetries of AIS girls are of unknown pathogenetic significance;

• current treatment at best is treating the morphologic and functional sequelae of AIS and not the cause of the disease; and

• whatever hypothesis or theory, several fundamental questions and facts of AIS need to be properly addressed and explained with 11 items listed.

## Pathogenesis - theories, hypotheses and concepts 2012

Several theories, hypotheses and concepts for AIS pathogenesis more recently implicating AIS as a systemic and/or multifactorial disorder, are providing hypotheses to test. [3-5,7]. These include:

• Relative anterior spinal overgrowth (RASO) [19].

***Sagittal spinal profile in fathers and mothers of AIS girls.*** In Utrecht, The Netherlands, Janssen *et al.*[20,21] tested the hypothesis that the familial trend of AIS is explained by the inheritance of the sagittal spinal profile, making the spine less resistant to rotatory forces. Using free-standing lateral spinal radiographs of 51 parent couples of girls with severe progressive AIS and 102 age-matched controls, they found that the fathers, but not the mothers had significantly flatter sagittal spinal profiles.

***Forceful restoration of the thoracolumbar lordosis to correct double major AIS.*** In Enschede, The Netherlands, Van Loon [22] reviewed factors in healthy children that can deform their spines.

***The use of growth in thoracolumbar lordotic intervention for the brace treatment of AIS.*** In Enschede, The Netherlands van Loon *et al.*[23] evaluated old and new concepts on the pathogenesis of AIS with respect to thoracolumbar lordotic intervention for brace treatment.

***Pelvic incidence and lordosis (anterior pelvic tilt).*** In Utrecht, The Netherlands, Janssen *et al.*[24] defined pelvic lordosis as the angle between the axis of the ischium and a line connecting the midpoint of the sacral endplate to the hip axis. Pelvic lordosis correlated strongly with pelvic incidence. Its role in spinal pathology deserves further investigation.

• Asynchronous spinal neuro-osseous growth [25,26].

***Typical and atypical AIS.*** In St Petersburg, Russia, Dudin and Pinchuk [27] presented a theory of AIS pathogenesis. Two forms of AIS are identified: typical AIS with convex-side axial vertebral rotation; and atypical scoliosis with concave-side axial vertebral rotation. The components of the theory are:

1) Growth under nervous and endocrine system control in pre-pubertal and pubertal periods differing in girls and boys, with “personal” programs in spinal cord and its bone-ligament-muscular sheath.

2) Non-conjugacy of growth (growth conflict) between spinal cord and its bone sheath – termed by others asynchronous, or uncoupled, neuro-osseous growth. This non-conjugacy of growth with –

a) excess length of vertebral column leads to convex-side vertebral rotation (typical AIS), and

a) decreased length of vertebral column leads to concave-side vertebral rotation (atypical AIS).

3) Formation of a scoliosis curve(s) involving Hueter-Volkmann effect and a vicious circle (Stokes),

• Thoracospinal concept [28].

• Dorsal shear forces and axial rotation instability [29].

***Pre-existing axial rotation of the normal spine.*** In an Invited Lecture, Castelein [30] in Utrecht, The Netherlands, reviewed their analysis of the rotational patterns of the normal growing and adult spine in relation to closure of the neurocentral junctions, organ anatomy, and the convexity of the curve in idiopathic scoliosis.

***Neurocentral junction (NCJ) in the normal growing spine.*** In Utrecht, The Netherlands, Schlosser *et al.*[31] evaluated the closure pattern and symmetry of left and right NCJs in the normal human growing spine in relation to pre-existing axial spinal rotation and the convexity of the curve in AIS.

• Flexural-torsional buckling from flexibility anisotropy [32].

• Biomechanical spinal growth modulation [33].

***Effects of carrying weight on posture.*** In Middlesborough, UK, Bettany-Saltikov and Cole [34] evaluated the effects of carrying weights (front packs, shoulder bags and hand held bags, each about 15% of body weight) on back shape and posture using an ISIS2 scanner on 25 university students. The shoulder and hand held bags produced postural deviations in all planes; these may cause stress and strain on the spine and lead to scoliosis curve progression.

***Lumbo-sacral-joint efforts during gait and Hueter-Vollkmann effect.*** In Montreal, Canada, Brussels, and Louvain, Belgium, Raison *et al.*[35] studied adolescents with left lumbar and thoracolumbar AIS and controls using an acquisition system involving body joint motion via optokinetic sensors and ground reaction forces via a treadmill fitted with force sensors. At L5-S1 subjects with severe AIS had higher medio-lateral forces than the controls which could lead to asymmetrical vertebral growth modulation and curve progression (Hueter-Volkmann effect).

***Growth-plate mechanobiology.*** In Montreal, Canada, Menard *et al.*[36] studying rat caudal vertebrae found dynamic loadings modulated growth with less damage to growth plates than static loading.

***Effect of torque on growth in caudal vertebrae.*** In Milwaukee, USA, Rizza *et al.*[37] using the rat tail, applied torsional loads to vertebrae that led to curvature in growth-plate morphology and remarkably increased growth-plate thickness.

***Osteoblasts, biomechanical stress and oestrogens.*** In Montreal, Canada, Moldovan *et al.*[38] in AIS subjects demonstrated that biomechanical stress and estradiol are involved in the expression of certain genes. Cultured osteoblasts subjected to biomechanical stress showed increased levels of NO, COX-2, OPN, and ATP levels in both control cells and AIS cells with significantly higher levels of NO and COX-2 in AIS cells.

• Biomechanical theory [39].

***Causative role of gait and standing at ease on right leg.*** In Lublin, Poland, Karski [40] outlined his theory that idiopathic scoliosis is connected with gait and persistent standing at ease on the right leg. Scoliosis, results from asymmetry of function proving the possibility of causative prophylaxis.

• Intervertebral disc disorder [41-47].

• Deforming three joint complex hypothesis [48].

• Motor control disorder [49-51].

***Hand grip strength.*** In Hong Kong, China, Yu *et al.*[52] fond that hand grip strength was lower than controls suggesting muscle dysfunction in AIS.

• Sensorimotor integration disorder & dystonia [51,53].

• Sensory integration disorder [54].

• Vestibular disorder [55].

***Semicircular canals.*** In Hong Kong, China, Chu *et al.*[56] used MRI to evaluate the morphology of semicircular canals (SSCs) in subjects with right thoracic AIS and controls. Significant differences in the shape of left, but not right, SSCs were found between AIS and controls. It was suggested that these morphological changes are likely to be related to subclinical postural, vestibular and proprioceptive dysfunctions in AIS subjects.

• Body spatial orientation disorder [57].

• Neurodevelopmental disorder [58].

• Systemic and metabolic disorders involving -

○ Platelet calmodulin [59,60].

○ Melatonin [61-64].

***Melatonin receptors.*** In Hong Kong, China Yim *et al.*[65] extended their previous research which showed that melatonin receptor 1B (MTNR1B) was not detected in osteoblasts of some AIS girls. In 41 AIS girls, while MTNT1A and MTNR1B were found in all, that of MTN1RB was lower, suggesting a quantitative rather than a qualitative difference linked to the pathogenesis of AIS.

○ Melatonin-signalling defect (MSD) [66,67].

○ Osteopontin (OPN) and soluble CD44 (sCD44). Azeddine *et al.*[68] and Moreau *et al.*[69] reported mean plasma OPN levels to be increased in:

• patients with idiopathic scoliosis, correlating significantly with curve severity, and

• “an asymptomatic at-risk group” (offspring born from at least one scoliotic parent); this finding, if confirmed, suggests predictive biomarkers and possibly a prodromal stage with the prospect of intervention early in deformity evolution.

In contrast, mean plasma levels of sCD44 were significantly lower in patients with Cobb angles of 45 degrees or more. Drawing on evidence from mouse models, it was concluded that OPN is essential to induce scoliosis formation and curve progression through interactions with CD44 receptors, *“….thus offering a first molecular concept to explain the pathomechanism leading to the asymmetrical growth of the spine in idiopathic scoliosis.”*[69].

Moreau and colleagues report that blood tests could be useful markers for the diagnosis of idiopathic scoliosis and the prognosis of curve progression: a functional scoliosis test [66-70], further refined recently using a more accurate technology called cellular dielectric spectroscopy [71]; and a biochemical scoliosis test using raised plasma OPN and lower sCD44 values. Moreau [72] states that OPN and sCD44 are not disease-specific but when observations of both are combined they become highly specific for idiopathic scoliosis. By binding free OPN, sCD44 can prevent OPN from triggering scoliosis or curve progression. Moreau considers that environmental factors could potentially affect the circulating levels of OPN in humans. With colleagues he is conducting tests to identify potentially useful therapeutic agents [72].

***High osteopontin (OPN).l levels and bone mineral density.*** In China and Montreal, Sun *et al.*[73] in 45 AIS girls demonstrated that low cortical bone mineral density in the distal radius is significantly associated with high OPN levels. It is stated that high osteopontin levels in plasma may reflect underlying abnormalities of bone mineralization in AIS subjects.

***PTPx and HSJ family members as disease-modifying factors in AIS.*** In Montreal, Canada, Elbakry *et al.*[74] stated that their previous research had found that AIS patients have a Gi protein signalling defect and high levels of circulating osteopontin (OPN). Here, on AIS and control osteoblasts, they investigated Protein Tyrosine Phosphatase x (PTPx) and HSJ-1 related family members to determine their potential contribution to OPN receptor activity. Bipedal PTPx knock-out mice were also evaluated. It was found that PTPx and HSJ-1 messenger RNA and protein levels were decreased in all 34 AIS patients compared with 17 controls. They concluded that PTPx and some of the HSJ family members have potential roles in AIS etiopathogenesis as disease-modifying factors exacerbating scoliosis development triggered by OPN.

○ Oestrogens [75,76].

***Oestrogen receptor 2 (ESR2) expression in back muscles.*** In Poznan, Poland, Rusin *et al.*[77] reported asymmetric ESR2 in deep paravertebral muscles more on the convexity than the concavity. With ESR2 convex/concave ratios of 1 or more, the ratio correlated with Cobb angle.

○ Leptin [7,76,78,79] (see 9.1.3)

***Leptin and bone mineral density.*** In Hong Kong, China, Tam *et al.*[80,81] in AIS and control girls, evaluated correlations between each of leptin and soluble leptin receptor (sOB-R) and volumetric bone mineral densities (vBMDs) of various bone compartments scanned at the distal radius using high resolution peripheral quantitative computed tomography (HR- pQCT). They stated that serum total leptin has an anabolic effect on BMD and suggested that AIS girls have an abnormal bone metabolic response to serum leptin. It is unclear whether this represents signalling dysregulation, or abnormal leptin bioavailability between bone compartments. (compact and trabecular).

***Leptin and cellular dysfunction.*** In Hong Kong, China, Tam *et al.*[82] reported that AIS girls have lower BMI, free leptin index (leptin/sOB-R), and higher sO-BR than controls. sOB-R expression correlated abnormally with each of fat content, BMI and leptin compared with controls. The authors speculate firstly, that AIS girls have cellular dysfunction that results in abnormal sOB-R expression which in turn may cause abnormal leptin bioavailability; and secondly, that sOB-R may predict curve progression.

***High central leptin activity in a mouse scoliosis model.*** In Nanjing, China, Wu *et al.*[83] reported results which indicated that high central leptin activity might increase the risk of developing a scoliosis in bipedal mice and contribute to scoliosis progression.

***Serum ghrelin levels.*** In France, de Gouzy *et al.*[84] found higher average levels of total serum ghrelin in AIS subjects compared with controls after adjusting for BMI. They suggested that ghrelin participates in the pathophysiology of AIS possibly involving ghrelin-like cell resistance to melatonin hormone.

***Neurohormonal regulation.*** In St Petersburg, Russia, Khaymina *et al.*[85] evaluated blood serum from 120 children age 8–15 years with AIS, Biotesting was performed on male Wistar rats with thoracic spinal cord transection. After 30 minutes and injecting the serum into the lumbar spinal canal, EMG activity of both hind limbs was recorded as a coefficient of movement disorder (CMD). The CMDs revealed scoliosis type by progression that are claimed to provide new opportunities for understanding the pathogenesis and methods of treatment for AIS.

• Osteopenia [86-88].

***Bone mineralization.*** In China, Sun *et al.*[89] evaluated cancellous bone from AIS and normal age-matched healthy subjects. Bone mineral density (quantitative microCT) and undecalcified histomorphometry were studied. The findings showed that AIS girls had lower bone mineralization than normals. It is suggested that an abnormality of bone matrix may play an important role in the etiopathogenesis of AIS.

***Bone quality.*** In Hong Kong, Yu *et al.*[90,91] reported that bone quality in osteopenic AIS girls was uniquely different from that of osteopenic non-AIS controls. Trabecular compartment alterations with osteopenia were only present in AIS girls, a finding that needs etiopathogenic assessment.

• Developmental instability & symmetry control dysfunction [92,93].

• Intrinsic growth plate asymmetry hypothesis [94,95].

• Multiple pathogenetic processes.

○ Double neuro-osseous theory [7,96].

○ Three components [10].

○ Four components [58,97,98].

***Is AIS under 20–30 degrees a chaotic dynamical system?*** In Lyon and Toulon, France, de Mauroy and Ginoux [99] outlined the relation of AIS to chaos.

## Some comments on theories, hypotheses and concepts 2012

### Attempts at integration of theories

None of the above theories entirely explain the pathogenesis of AIS [3-5,7,10,68]. In recent surveys of these theories for AIS [3-5,7,10,98], integration is attempted by involving interacting pathomechanisms. In addition to predisposing factors [3,6], there is a view that there are two other pathogenic processes for idiopathic scoliosis namely, initiating (or inducing), and those that cause curve progression [3,100]. There is evidence that curve progression, which increases during the curve acceleration phase [101], increases through disc wedging during the rapid growth spurt with progressive vertebral wedging occurring later [45,46]; and that in scoliosis the simultaneous occurrence of vertebral displacement in 3-D, rather than a specific single disturbance of any one of the three planes, triggers development of the deformity [102].

***Skeletal-size-for-age in single thoracic and single lumbar AIS.*** In Hong Kong and Nanjing, China, Liu *et al.*[103] compared anthropometric parameters of girls with single thoracic and single lumbar AIS, each against 3914 healthy girls. Compared with healthy girls, thoracic and lumbar AIS, girls had higher corrected standing and sitting height, arm span and leg length. Compared between thoracic and lumbar AIS, corrected standing and sitting height were not significantly different. Thoracic AIS girls were leaner than healthy girls.

***Abnormal skeletal growth patterns.*** In Hong Kong, China, Yim *et al.*[104] in girls age 12–16 years evaluated growth of two AIS groups (severe and moderate scoliosis) and control girls. In a cross-sectional study, severe AIS at age 12 started with a shorter arm span than moderate AIS and controls, but overtook at 14–16 years. In the longitudinal study from 12–16 years, moderate AIS had longer arm span than controls with similar growth rate. Severe AIS had a higher growth rate than moderate AIS. It is concluded that the growth spurt during puberty is a key factor associated with pathogenesis and curve progression in AIS. It is suggested that arm span growth could be used to predict severe scoliosis in AIS.

***Height velocity curves.*** In Tokyo, Japan, Chazono *et al.*[105] calculated peak height velocity (PHV) on 20 skeletally immature girls with idiopathic scoliosis with a follow-up period of 5.2 years. The findings were compared with standard data from unaffected girls. The findings show that while the PHV was higher and the growth period shorter in IS girls than in unaffected girls. It was suggested that curve progression is associated with the magnitude of PHV. and duration of the growth process.

### RASO phenomenon and asynchronous spinal neuro-osseous growth concept

Relative anterior spinal overgrowth (RASO) is established as a sagittal plane phenomenon associated with scoliosis. Its etiologic mechanisms are unknown but curve progression is thought to involve the Hueter-Volkmann effect [33]. Some workers adduce evidence that RASO results from a spinal neuro-osseous growth disorder – variously termed *vertebra-neural* (Roth, van Loon), *uncoupled (*Porter), and *asynchronous*[25] - of unknown etiology. Without riders these sagittal plane theories, like other biomechanical theories, do not accommodate -

• normal trunk bilateral asymmetry [96,106,107],

• normal thoracic spinal axial vertebral rotation [108],

• bilateral skeletal asymmetries of AIS [7],

• some bilateral neural [50,51] and vestibular [55] asymmetries, and

• the systemic and metabolic disorders associated with AIS.

The riders are secondary effects (epiphenomena) [3] and/or multiple factors possibly of different weight in individuals with similar deformities.

#### Posture – gender and age-specific features

In Novosibirsk, Russia, Sarnadskiy [109] reported on 33,000 children and adolescents from 5–17 years who were screened using computed optical topography. The most significant postural differences between boys and girls were in the sagittal plane. In the transverse plane, from the age of 5 years, the trunk twists in a clockwise direction in boys and girls. By the age of 17 years, the clockwise twisting is preserved in boys, but in girls counter-clockwise twisting is prevalent. These twistings are novel phenomena that need investigation.

#### Classification of postural and spinal disorders

In Novosibirsk, Russia, Sarnadskiy [110] presented a classification of postural and spinal deformities based on experience since 1994 of using computed optical topography for spinal deformities.

#### Longitudinal evolution of back shape in three planes

In Pern, Russia, Pecherskiy *et al.*[111] using computed optical topography studied the “preclinical form” (Dudin and Pinchuk) or “dark zone” (Bagnall) of AIS, by evaluating back shape in schoolchildren on three occasions. Seven morphological groups were identified with only ¼ children maintaining their group and others “migrating” between groups.

#### Faulty body posture and bilateral asymmetry

In Lodz, Poland, Kluszczynski and Czernicki [112] reported a longitudinal study of 100 children and adolescents examined for body posture in 1997 and again in 2007. In the girls during pubertal growth, thoracic kyphosis decreased as lumbar lordosis increased, with an opposite tendency in boys. Back asymmetry frequency increased in both sexes, left thoracolumbar, right thoracic and left lumbar, as did pelvic asymmetry and functional asymmetry of the right lower limb.

#### Posterior trunk asymmetry

In Poznan, Poland, Stolinski and Kotwicki [113] reported the use of a Bunnell Scoliometer to measure the angle of trunk rotation (ATR) at three levels (thoracic, main thoracic and lumbar) in a standing forward bending position in 9,500 Primary schoolchildren age 7–10 years

#### Anterior trunk asymmetry

In Poznan, Poland, Stolinski *et al.*[114] reported a new parameter, *anterior trunk asymmetry index* conducted on 50 schoolchildren age 6–7 years.

#### Transient upper arm length asymmetry associated with right thoracic AIS (RT-AIS)

In Nottingham, UK, Burwell *et al.*[115] in right-handed girls with RT-AIS found that upper arm length asymmetry (right-*minus*-left) 1) regressed negatively with each of age and years after estimated menarcheal age, 2) was greater than in healthy girls, and 3) correlated significantly with Cobb angle and apical vertebral rotation. The findings have relevance to RT-AIS pathogenesis, and the use of arm span to measure skeletal overgrowth in AIS.

#### Leg length discrepancy (LLD) in scoliosis patients

In Pescara, Italy, D’Amico *et al.*[116] using an opto-electronic system on 143 scoliotic patients with a mean LLD of 10 mm, confirmed a previous study that demonstrated the efficacy of under-foot wedge. in LLD correction.

### Sensory and sensorimotor integration disorder and dystonia

There is increasing evidence for the possibility of an underlying neurological disorder for AIS; this research needs to ne extended. The findings relate to sensory integration [54], sensorimotor integration and dystonia [50,51,53], spinal cord [25,26], brain stem [9], motor control [49], motor cortex [50], supplementary motor area [51], brain white matter [117] as part of the *Human Connectome*[118], cerebral cortex as a whole [119] and vestibular system [55]. The two papers by Domenech *et al.* focus attention on bilateral asymmetric activity respectively of motor cortex [50] and supplementary motor area [SMA, 51] as pathomechanisms for idiopathic scoliosis (IS). Reviewing the SMA paper [51] Benoist [120] writes:

*“These findings support the hypothesis that a sensorimotor integration underlies the pathogenesis of IS. In addition, as suggested by the authors, these abnormal MRI findings may represent a biomarker of IS disease and open the way to novel therapeutic targets. This paper, winner of the EuroSpine Full Paper Award for 2010, has limitations, which have been summarized in a Reviewer’s Comment by Freeman*[121]*.”*

#### Somatosensory and motor evoked potentials

Huber and Rogala [122] in 136 AIS patients before treatment found asymmetrical deficits in both afferent (SEP) and efferent (MEP) transmissions especially at the level of the apical vertebra of the main curvature, but also in 45/65 patients in the brain stem. These findings were related to structural changes in spinal cord and brain stem.

#### EEGs and peculiarities of brain functioning in AIS

In St Petersburg, Russia, Pinchuk *et al.*[123] using an original analysis of EEGs revealed a shift of bioelectric activity focus to the left hemisphere in patients with AIS. and more with increasing curve severity that may be prognostic for AIS. This interhemispheric asymmetry was not observed in healthy children 7–14 years, but was found in healthy adolescents at 15–17 years and in adults. In AIS, it represents an earlier maturation than in healthy subjects with the brain playing a more important role in right- than left-sided AIS. They suggest the underlying mechanism is an “overstrain of the central nervous system adaptation-compensation mechanisms during the pubertal period.”

#### Cerebral cortical thickness

In Hong Kong, China, Chu *et al.*[124] reported findings implying a different thinning pattern of the cerebral cortex during adolescence in patients with AIS; this may be primary (etiopathogenetic) or secondary to the development of the scoliosis.

#### Postural control

In Poznan, Poland, Wiernicka *et al.*[125] evaluated static and dynamic postural control to body mass loading (BML) during single and double lower limb stance on respectively a force platform and rolling rocker board. In 30 girls with thoracolumbar idiopathic scoliosis (IS), foot arch shape differently controls static and dynamic balance. compared with 34 healthy girls. IS girls with flattening of the foot and those with foot arch preserved during BML had respectively higher and lower sway pathways than controls.

#### Back muscle bioelectric activity

In St Petersburg, Russia, Syngayevskaya *et al.*[126] studied back muscle bioelectric activity in children 10–15 years of age. They found paravertebral muscle activity at the curve apex was higher on the convexity and, in the distal part, higher on the concavity compared with the opposite side. H-reflex and M-responses were studied in relation to curve progression.

### Lessons from brain research - brain in a box

The diversity between the above theories reveals current ignorance about the causation of AIS indicating more integrated knowledge is needed. Like brain research [127,128], most AIS workers are focused on their own corner. In brain research, which generates about 60,000 papers per year, one team in Europe is planning to integrate these discoveries and achieve a comprehensive understanding of the brain; this would be done by building unifying computer models (‘brain in a box’) for which one billion euros is being sought in 2012 from the European Union’s new decade-long ‘flagship’ initiatives [127] (see Conclusions).

### Grand unifying theory for AIS scoliogeny - brain and scoliosis in a box?

In AIS, the possible creation of a network approach to the pathogenesis by constructing AIS *diseasomes*[129] was suggested [18]. The attainment of a grand unifying theory for adolescent idiopathic scoliogeny seems unlikely at present. Finite element analysis of spinal models of AIS pathogenesis (‘scoliosis in a box’) has provided for the testing of specific questions, recently in connection with how accelerated growth profiles may increase scoliosis progression and pose a progressive risk factor [130]. Building a unifying computer model for AIS pathogenesis might be considered using supercomputers with ever increasing memory (‘brain and scoliosis in a box’).

### The biological Higgs – aging, AIS and scoliogeny

Biologists have recently pondered what fundamental discoveries might match the excitement of the Higgs boson, the so-called ‘God Particle’ [131]. Some scientists consider that the ability to slow aging would address Higgs-like fundamental questions about human life. AIS, we suggest, may be viewed as a disorder of aging processes during development. So questions about what pathways control aging - highly unlikely to result from a unitary cause [131,132] - and some age-related diseases, may be relevant to the scoliogeny of AIS (see 9.1.1).

## Genetics, epigenetics and AIS scoliogeny

After mentioning genetics we focus on epigenetics and how it might relate to AIS scoliogeny.

## Genetics and the genetic variant hypothesis for complex disease 2012

### Etiology of AIS is poorly understood

Twin studies and observations of family aggregation have revealed significant genetic contributions to idiopathic scoliosis that position AIS among other common disease or complex traits with high heritability [133]. However, despite many investigations, the underlying etiology of idiopathic scoliosis is poorly understood [6,134].

### Position for AIS

Based on gene-linkage studies, candidate gene approach and genome-wide association studies, Wang *et al.*[5] summarized the then position as follows:

*“Recent evidence not only suggests that genetic factors play an important role in the etiology of AIS but also revealed the considerable heterogeneity. Although the continuation of the search for the exact genetic factors is inevitable, it is also very important to look at the functional and biological aspects to allow better understanding of the etiopathogenesis if AIS*.*”*

#### An axial developmental gene (VANGL1) harbours a mutation causing idiopathic scoliosis

In the USA, Wise *et al.*[135] used next generation sequencing to discover a gene that harbours a mutation causing IS in a three generation family. An excess of mutations of this gene was found in subsequent series of IS patients compared with controls. They comment that *VANGL1* participates in a biochemical pathway critical in axial development, specifically controlling neural tube development and spinal cord directionality.

#### New susceptibility locus for AIS

In Japan, Takahashi *et al.*[136] through a generation wide and replication study in Japanese, identified SNPs near LBX1 significantly associated with susceptibility of AIS.

#### Gene profiles, cellular and biochemical endophenotypes of idiopathic scoliosis (IS)

In Montreal, Canada, Gorman *et al.*[137] aiming \to discover expressed genes among their three endophenotypes (groups 1–3), used a microarray analysis on osteoblasts and peripheral blood mononuclear cells (30 patients with IS, 10 controls), analyzing 38,000 genes. It was found that generally, the expression profiles for groups 2 & 3 were similar to each other in contrast to that of group 1, reflecting functional responses to osteopontin.

#### CHD7 polymorphism in a Central European sample

In Poznan, Poland, Janusz *et al.*[138] stated that in North America, single SNPs of the *CHD7* gene have been shown to be associated with idiopathic scoliosis (IS) susceptibility. In 44 females including IS and controls from Central Europe, no association was found between the *CHD7* gene and either susceptibility to IS, or its severity.

#### Gene polymorphisms and AIS susceptibility

In Nanjing, China, Zhou *et al.*[139] stated that genome wide association studies suggested that gene polymorphisms of *IL-17RC, CHL1, DSCAM,* CNTNAP2 are associated with AIS. In a Chinese Han population with AIS, they reported that while *IL-17RC* may be a susceptibility gene for AIS, *CHL1, DSCAM, CNTNAP2* genes were not associated with AIS.

#### Gene expression, biomechanical stress and estradiol

In Montreal, Canada, Moldovan *et al.*[38] demonstrated that biomechanical stress and estradiol are involved in the expression of certain genes in osteoblasts from AIS subjects which could impact curve progression. Using microarray analysis, 86 genes were expressed at relatively higher levels in AIS osteoblasts compared with controls, while 59 genes were expressed at lower levels. The genes are reported to be involved in bone metabolism and embryonic development suggesting various gene interactions and pathways in AIS pathogenesis. The research provided a previously unrecognised list of candidate genes for AIS.

#### *Selection of subjects for genetic association studies for idiopathic scoliosis*/

In Poznan, Poland, Harasymczuk *et al.*[140] after examining English Language articles from 2001-2011, stated that most to-date were not based on common inclusion criteria which is needed.

#### Large multiplex French family with idiopathic scoliosis (IS)

In Montreal, Canada, Moldovan *et al.*[141] evaluated a large multiplex French family with IS. Within two chromosomal loci linked to IS susceptibility, several novel variants were found; two of these variants co-segregate perfectly with the disease.

### AIS Prognostic Test (AIS-PT, Scoliscore)

Genetic profiling using 53 single nucleotide polymorphisms (SNPs), gene-to-gene interactions, and the patient’s initial Cobb angle measurement, produced a score ranging from 1–200 predicting scoliosis curve progression [142-145] and provided a scientific basis for the *AIS Prognostic Test (Scoliscore, Axial Biotech, Inc.).*

#### Clinical value of an intermediate risk score with AIS Prognostic test (AIS-PT)

In the USA, Ward *et al.*[146] concluded that within the intermediate score range, individual risks vary tremendously (>20 fold) based on the actual score observed.

#### AIS Prognostic Test testing of patients with advanced Cobb angle provides further validation of the test algorithm

In the USA, Nelson *et al.*[147] showed that patients with advanced Cobb angle showed elevated *Ptognostic Test* values. None of the patients who progressed to severe curves had low risk scores.

#### Prognostic testing cannot be applied to all racial groups without modification

In the USA, Ward [148] compared DNA samples from AIS patients with controls from Asian and African ancestry. DNA markers were related to AIS progression and vary by race. Differences in gene variants will translate into differences in the underlying chemistry of AIS causing different clinical expression.

#### Prognostic Test scores are higher in patients who fail orthotic treatment for AIS

In the USA, Ogilvie *et al.*[149] stated that although the DNA-based AIS *Prognostic Test* was not designed to predict brace success or failure, the data show that underlying genetic factors may play an important role in response to bracing.

#### The use of 3D spinal parameters to differentiate between progressive and non-progressive AIS curves at the first visit

In Canada, Nault *et al.*[150] at the first clinic visit, confirmed there are morphological differences between progressive and non-progressive AIS curves; these include axial rotation, hypokyphosis, plane of maximal curvature and torsion of the spine. The findings are claimed to underscore the importance of the torsional aspect of the deformity occurring at the junctional zone below the main curve. Wedging does not seem to be related to progression at this early stage.

### Genetic variant hypothesis and non-genetic factors for complex disease

Butcher and Beck [151] write:

“A spate of high-powered genome-wide association studies (GWAS) have recently identified numerous single-nucleotide polymorphisms (SNPs) robustly linked with complex disease. Despite interrogating the majority of common human variation, these SNPs only account for a small proportion of the phenotypic variance, which suggests genetic factors are acting in concert with non-genetic factors. Although environmental measures are logical covariants for genotype-phenotype investigations, another non-genetic intermediary exists, epigenetics.”

## Epigenetics - perspective for AIS

### Definition and epigenetic modifications

Epigenetics is generally defined as information heritable during cell division but not contained within the DNA sequence itself, termed *epigenetic modifications*[18,152,153].

There are the three major ways organisms alter their DNAs inherited messages [153,154]:

• enzymes methylate DNA to modulate transcription;

• histone modifications and nucleosome positioning to induce or repress target sequences; and

• non-coding small RNAs (including microRNAs and short interfering RNAs) which attach themselves to messenger RNA to modify the expression of specific genes.

### DNA methylation

According to Talens *et al.*[155]:

“DNA methylation may be the most suitable epigenetic mark for large-scale epidemiological studies, since methyl groups are covalently bound to CpG dinucleotides and are not lost during routine DNA extraction, unlike histone modifications. This opens the possibility of exploiting existing DNA biobanks for research purposes, to discover epigenetic risk factors for complex disease.”

### Epigenetics at the epicenter of modern medicine

Epigenetics evaluates factors concerned with gene expression in relation to environment, disease, normal development and aging, with a complex regulation across the genome during the first decade of life [16-18].

Feinberg [13] writes:

*“Epigenetics, the study of non-DNA sequence-related heredity, is at the epicenter of modern medicine because it can help to explain the relationship between an individual’s* genetic background, the environment, aging, and disease…*”*

### Environmental factors and AIS

In the last 20 years, sporadic reports have suggested environmental factors are involved in the etiopathogenesis and phenotypic expression in some subjects with AIS. We review the evidence elsewhere [18].

#### Truncal asymmetry and maternal age at birth

In Athens, Greece, Grivas *et al.*[156] reported evidence from schoolchildren that maternal age through epigenetic mechanisms, may influence the occurrence of truncal asymmetry during growth in males more than females.

### Hypothesis of developmental instability for scoliosis

Speculation that genetic and environmental factors are involved the etiopathogenesis of idiopathic scoliosis [157,158] was further developed by Goldberg and colleagues [92,93] who suggested that scoliosis is caused by environmental stress causing developmental instability:

### Heated indoor swimming pools, infants and AIS as a delayed epigenetic effect

**Figure 1:**
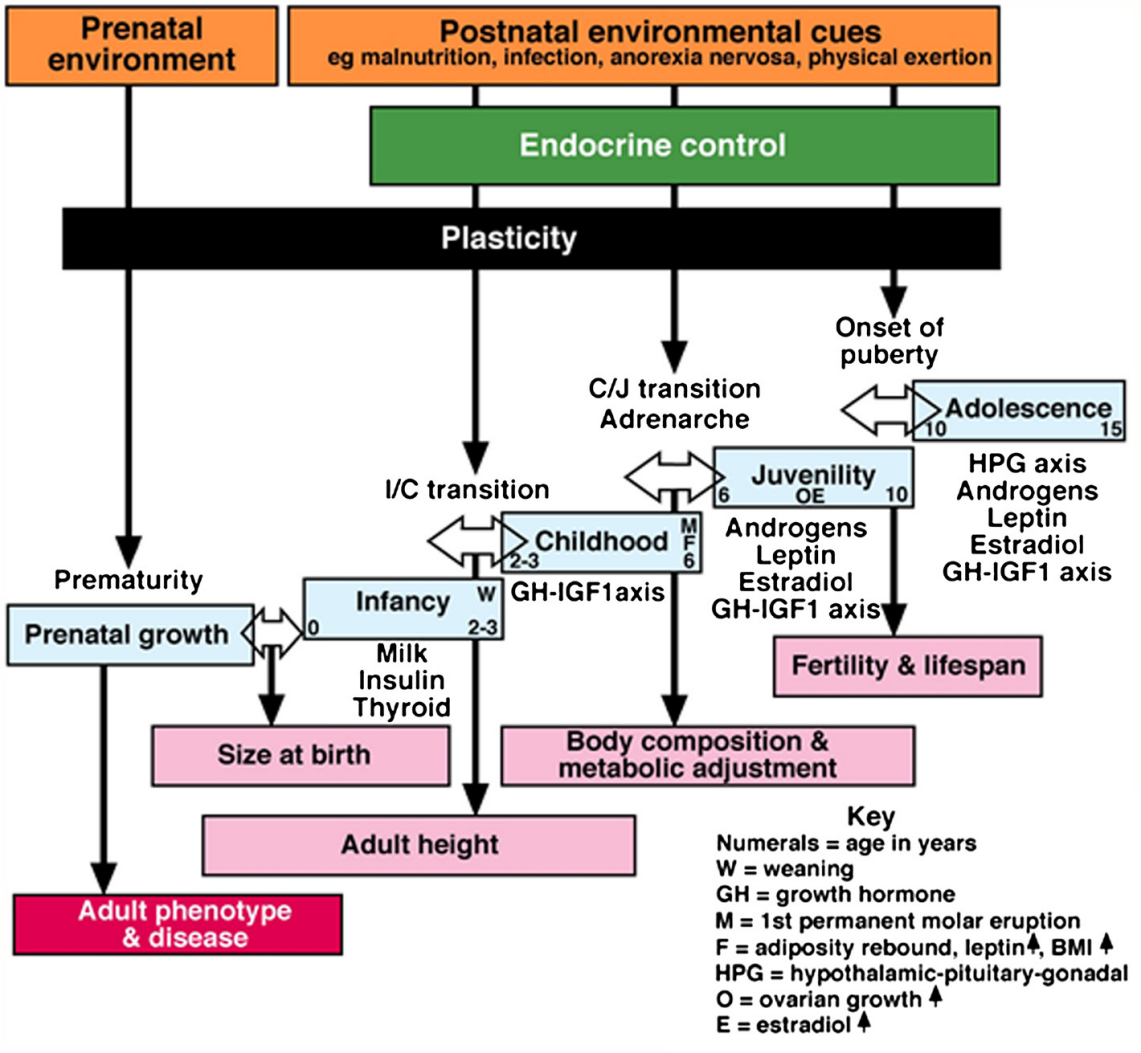
**Pre-adult periods of adaptive plasticity in the transition between life history phases (from Burwell *et al. *[**[163]**] modified from Hochberg *et al. *[**[11]**]).**

McMaster *et al.*[159,160] reported a statistically significant correlation between the introduction of infants to heated indoor swimming pools and the development of AIS. A neurogenic hypothesis was formulated to explain how neurotoxins produced by chlorine may act on the infant’s immature central nervous system with an implication of the brain’s barrier and cerebral spinal fluid being involved. The delayed epigenetic effects with the bony trunk deformity of AIS do not become evident until adolescence [161]. There may be many such environmental factors acting in the first year of life to initiate AIS and differing around the world (see Figure 1). Whatever the effects, the neurotoxic products may have on the immature brain, the process of puberty with its increased growth velocity is suggested to play a role in the delayed phenotypic expression of AIS [101,162].

Elsewhere we outline and comment on etiopathogenetic concepts as they may relate to normal spine development and AIS pathogenesis [18].

## Human development – normal life history phases and epigenetics

### Developmental plasticity and epigenetic programming

Reviewing child health, developmental plasticity and epigenetic programming, Hochberg *et al.*[11] describe how transition between life-history phases defines pre-adult phases of predictive adaptive responses (plasticity) (Figure 1).

• Infancy-to-childhood (I/C) transition is associated with GH-IGFI axis activation that affects adult height;

• Childhood-to-juvenility (C/J) transition affects body composition, adiposity rebound and circulating leptin levels.

### Life history phases, epigenetics and AIS

We applied this concept speculatively to AIS pathogenesis in susceptible girls [163].

#### Body composition and nutritional status

In Katowice, Poland, Matusik *et al.*[164] evaluated nutritional status of children and adolescents with idiopathic scoliosis, early onset (n = 25) and late onset (n = 34). Overweight and obesity had the same prevalence as the normal population, Nutritional abnormalities were more common in boys. Early onset patients are thinner than the late-onset group.

## Environment, non-communicable diseases

### Idiopathic chronic non-communicable diseases (NCDs)

#### Types of NCDs, risk factors and prevention

**Figure 2:**
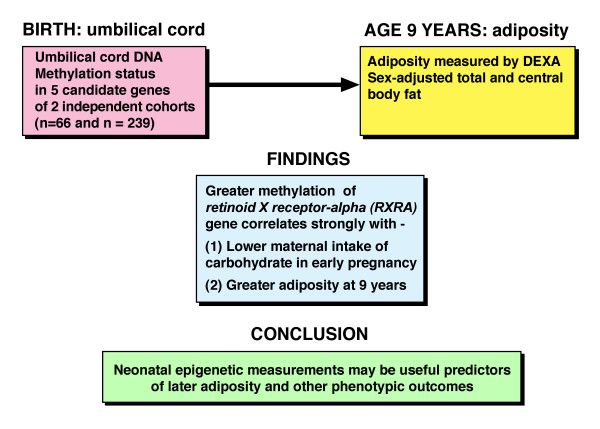
**Diagram summarizing the study of Godfrey *et al. *[**[170]**] who state that genome-wide association studies showed fixed genetic variation makes little contribution to the risk of obesity, heart disease, or diabetes.** Godfrey *et al.* conclude that their findings “… raise the possibility that the developmental environment component may be equally or more important.” We suggest that after consideration of the selection of appropriate candidate genes, umbilical cords may be stored and the children later evaluated for trunk deformity of AIS and normal trunk asymmetry [96,106,107].

Common chronic NCDs include obesity, diabetes, cardiovascular disease, respiratory disease, cancer and schizophrenia attributed to genetic and environmental factors [12] with a pathogenetic link being defined between obesity and cancer [165]. Risk factors for NCDs include age, unhealthy diet, smoking, alcohol abuse, chemicals, and lack of physical activity [131,161,166,167], the last possibly by impairing each of autophagy, mitochondrial upgrade and energy production [168]. In studying the prevention of NCDs, there is a move away from adults to mother, father, pregnancy and child [169], with recent research revealing a link between gene promoter methylation in umbilical cord tissue and later adiposity (Figure 2) [170].

#### AIS as an NCD?

Although AIS is not regarded as an NCD, like them and particularly like obesity, it is often associated with whole organism but opposite metabolic phenomena, namely lower body mass index (BMI) [171,172], lower circulating leptin levels [78,79], and other systemic disorders [59-64,66-72].

#### AIS, obesity, hypothalamic sensitivity and resistance to leptin

In the leptin-hypothalamic-sympathetic nervous system (LHS) concept for AIS pathogenesis of girls, scoliosis initiation is attributed to increased hypothalamic sensitivity to leptin [for central leptin activity see [83,173,174] with sympathoactivation becoming asymmetric as an adverse hormetic effect [7,175,176]; this places AIS with lower BMI at the opposite end of the spectrum from the central leptin resistance concept for obesity [7]. The putative asymmetric sympathoactivation is viewed as one mechanism initiating the costo-vertebral asymmetry of right thoracic AIS in girls [7,28] that produces the major phenotypic abnormality, namely the scoliosis of a whole organism (systemic) disorder.

## Methodological application of epigenetics to epidemiological studies

Some methods in DNA methylation profiling are reported by Zuo *et al.*[177]. The application of epigenetic methods to epidemiological studies is discussed here in relation to some other diseases, and then how these methods might be applied to AIS.

## Applying epigenetic methods to some other diseases

Mazzio and Soliman [17] write:

“One of the greatest challenges in the study of epigenetics as it relates to disease is the enormous diversity of proteins, histone modifications and DNA methylation patterns associated with each unique maladaptive phenotype. This is further complicated by a limitless combination of environmental cues that could alter the epigenome of specific cell types, tissues, organs and systems.”

We examine here what has been found epigenetically for -

• Silver- Russell syndrome,

• Idiopathic chronic non-communicable diseases (NCDs) some of which afflict children,

### Silver-Russell syndrome (SRS)

Silver-Russell syndrome is a clinically and genetically heterogeneous congenital disorder characterized by severe growth retardation [178] with bilateral skeletal asymmetry and genital anomalies. Bruce *et al.*[179] found a dose–response relationship between the degree of H19 hypomethylation and phenotype severity in SRS. The association between severe H19 hypomethylation and specific anomalies of the spine, elbows, hands and feet, and genital defects, was shown for the first time.

### Idiopathic non-communicable diseases (NCDs)

#### Fixed genomic variation

According to Godfrey *et al.*[170]:

“Fixed genomic variation explains only a small proportion of the risk of adiposity in animal models; maternal diet alters offspring body composition, accompanied by epigenetic changes in metabolic control genes. Little was known about whether such processes operate in humans.”

#### Gene promoter methylation in umbilical cord tissue at birth and later adiposity

Using Sequenom MassARRAY, Godfrey *et al.*[170] measured the methylation status of 68 CpGs 5′ from five candidate genes in umbilical cord tissue DNA of healthy neonates (Figure 2). Methylation varied greatly at particular CpGs. They related methylation status to maternal pregnancy diet and to child’s adiposity at age 9 years (by dual energy X-ray absorptiometry) with replication established in a second independent cohort. They concluded that: (1) a substantial component of metabolic disease risk has a prenatal developmental basis, and (2) perinatal epigenetic analysis may have utility in identifying individual vulnerability to later obesity and metabolic disease.

## Suggestions for applying epigenetic methods to AIS etiopathogenesis

### Methylation of candidate genes – cross-sectional study

As for Silver-Russell syndrome [178,179], we suggest that the possible selection of appropriate candidate genes and evaluating their methylation status in relation to physical and other characteristics of AIS subjects be considered. Familial and sporadic AIS might be evaluated.

### Methylation of DNA in umbilical cord tissue at birth and later AIS – longitudinal study

As for adiposity (Figure 2) [170], the stored umbilical cords of children who later show trunk distortion [96,106,107] and AIS deformity may be evaluated for their DNA methylation status, with familial and sporadic AIS again evaluated.

### Methylation in buccal smear DNA and later AIS – longitudinal study

An evaluation might be considered using buccal smear DNA starting, say at 5 years, with familial and sporadic AIS being evaluated.

## The future – memorandum of understanding for AIS scoliogenic research?

We suggest consideration be given to forming a group to work out details of a possible international collaborative effort for epigenetic scoliogenic research on AIS, perhaps initially preparing a Memorandum of Understanding.

## Conclusions

Extracts of the selected AIS scoliogeny papers presented at the 2012 IRSSD and SRS Meetings and arranged in a review article [1] -

• reveal where progress is currently being made, and

• suggest fields where the focus is becoming clearer, and/or needs enlarging.

The latter include includes:

1. Preclinical form, dark zone and prodromal stage of AIS.

2. Human development and skeletal growth.

3. Back shape, back muscle activity, posture and gait.

4. Radiology.

5. Central nervous system, brain, vestibular system and spinal cord.

6. Hormones and receptors, including oestrogens, melatonin, leptin, ghrelin and neurohormonal regulation.

7. Melatonin receptors.

8. Melatonin-signalling dysfunction.

9. Osteopontin, CD44, PTPx, HSJ and chaperone molecules.

10. Osteoblasts, biomechanical stress and oestrogens.

11. Bone matrix, mineralization and osteopenia.

12. Bilateral skeletal asymmetries.

13. Simulation.

14. Genetics.

15. Epigenetics.

16. Prognosis.

17. Theory refinement and formulation.

18. As for brain research [127], future work on AIS etiopathogenesis requires research at different levels, integration of information, simulation and collaboration [180]. The European Union announced in January 2013 euros 500 million (US$670 million) for the http://Human Brain Project (2013-2023) [181], with the goal of drawing together all existing knowledge of the human brain employing supercomputer-based models and simulations to reconstruct the brain. In the USA. a decade-long programme is planned to examine brain function aiming to build a comprehensive activity map, and seeking to do for the brain what the http://Human Genome Project did for http://genetics[182,183].

19. In AIS, the ultimate hope is to prevent the occurrence or progression of the spinal deformity with non-invasive treatment, possibly medical. This might be attained by personalised polymechanistic preventive therapy targeting the appropriate etiology, or etiopathogenetic pathways, to avoid fusion and maintain spinal mobility.

## Competing interests

The authors declare that they have no competing interests.

## Authors’ contribution

The original “Whither” article was written by GB and presented at IRSSD by PD. JC initiated this review of the selected scoliogenic presentations at the 2012 IRSSD Meeting to which the 2012 SRS Meeting was added by GB. Copies of the Final SRS Program were given to GB by Mrs MJ McMaster and Dr NS Harshaavardhana. The selection of abstracts for inclusion was made by GB and discussed with JC, PD. AM and TG. All authors contributed their professional skills to the inclusions of the text. All authors have read and approved the final manuscript.
